# Princeton RAtlas: A Common Coordinate Framework for Fully cleared, Whole *Rattus norvegicus* Brains

**DOI:** 10.21769/BioProtoc.4854

**Published:** 2023-10-20

**Authors:** Emily Jane Dennis, Peter Bibawi, Zahra M. Dhanerawala, Laura A. Lynch, Samuel S.-H. Wang, Carlos D. Brody

**Affiliations:** 1 Princeton Neuroscience Institute, Princeton University, Princeton, New Jersey, USA; 2 Janelia Research Campus, Howard Hughes Medical Institute, Ashburn, Virginia, USA; 3 Howard Hughes Medical Institute, Princeton University, Princeton, USA; 4 Neurology Department, University of Pennsylvania, Philadelphia, Pennsylvania, USA; 5 Washington University School of Medicine, St. Louis, Missouri, USA

**Keywords:** Tissue clearing, Light-sheet imaging, Rattus norvegicus, Image registration, iDISCO, uDISCO, Rat, Neuroscience

## Abstract

Whole-brain clearing and imaging methods are becoming more common in mice but have yet to become standard in rats, at least partially due to inadequate clearing from most available protocols. Here, we build on recent mouse-tissue clearing and light-sheet imaging methods and develop and adapt them to rats. We first used cleared rat brains to create an open-source, 3D rat atlas at 25 μm resolution. We then registered and imported other existing labeled volumes and made all of the code and data available for the community (https://github.com/emilyjanedennis/PRA) to further enable modern, whole-brain neuroscience in the rat.

Key features

• This protocol adapts iDISCO (Renier et al., 2014) and uDISCO (Pan et al., 2016) tissue-clearing techniques to consistently clear rat brains.

• This protocol also decreases the number of working hours per day to fit in an 8 h workday.

Graphical overview

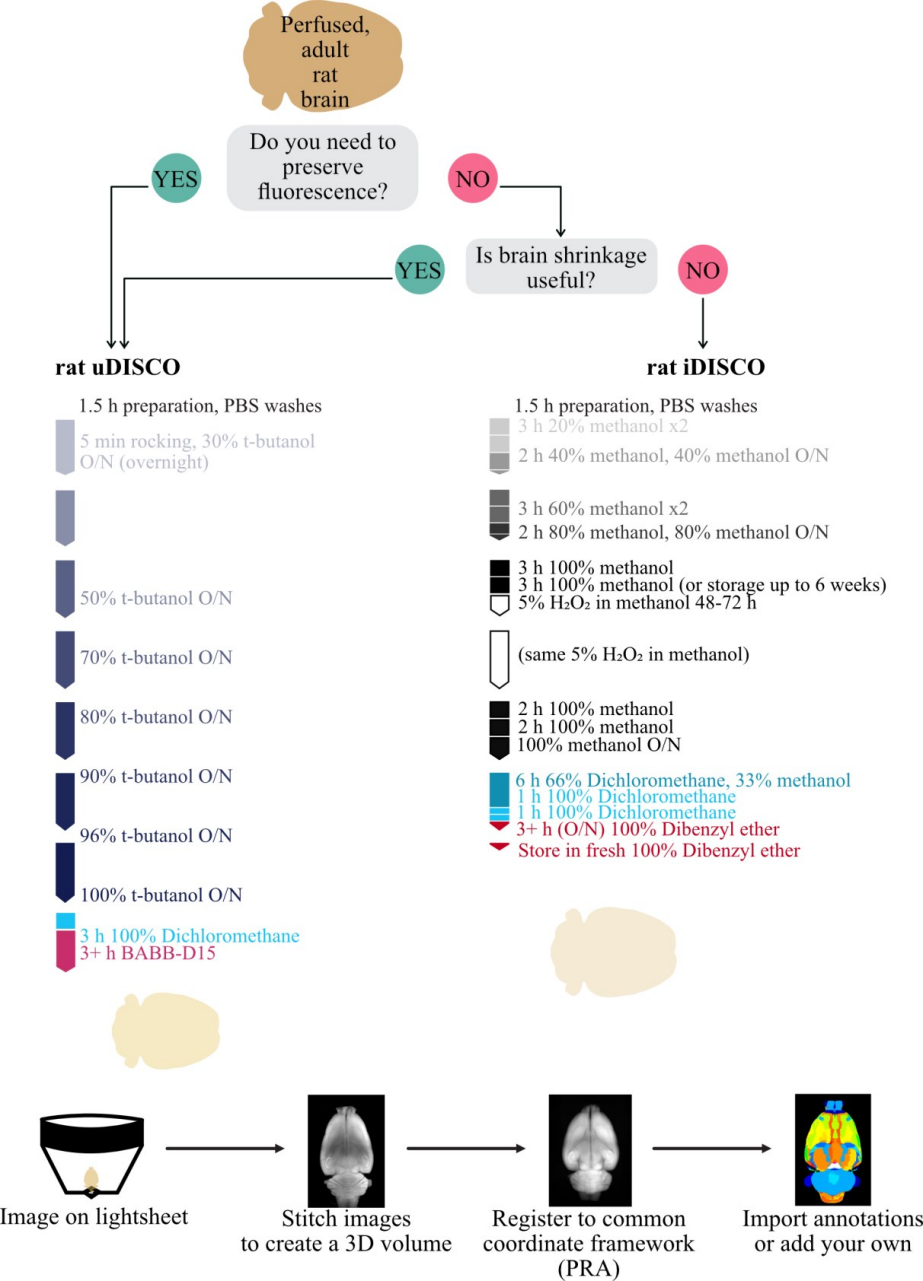

## Background

Recent developments in tissue clearing and imaging allow us to gather whole-brain connectivity in intact brains, together with classic neuronal tracing techniques. Though tissue fixation and preparation predated neuroscience, its use was refined by Golgi and further modified and popularized by Ramón y Cajal (1911). To this day, after experiments, neuroscientists often remove the brain and slice it into sections that are then mounted to microscope slides for imaging. This allows for both post-hoc verification of experimental targeting and, if paired with staining or immunohistochemistry, learning about the tissue. However, this process is hard to automate and time consuming: each step of fixation, removal, slicing, and imaging for each slice can take hours. Additional immunohistochemistry or staining can take weeks. To decrease the amount of labor required to process each brain, researchers will frequently only keep a subset of the brain slices near the relevant regions of study. Often this is more than sufficient, but this approach can easily miss potentially relevant information in the discarded sections, and sectioning itself can introduce deformations and aberrations to data that are difficult to identify post-hoc. To address this issue, several groups have developed methods to clear the lipids and pigments from whole brains, allowing for imaging through the entire tissue without the need for sectioning and often dramatically decreasing imaging time.

Building on advances from the early 1900s, tissue clearing has experienced a resurgence in neuroscience over the last 20 years. Methods like CUBIC (Susaki et al., 2015), CLARITY (Chung and Deisseroth, 2013), FluoClearBABB (Schwarz et al., 2015), and the DISCO methods (Renier et al., 2014; Pan et al., 2016) have become popular with researchers studying mice (see Molbay et al., 2021 for a review) yet those of us studying rats have not yet fully embraced this new methodology. One major impediment for broad acceptance of these methods in larger-brained animals is that existing clearing techniques do not consistently produce fully cleared, intact brains, prohibiting the study of subcortical structures in whole brains and decreasing their utility over slice histology. However, several advances have been made: Woo and colleagues nearly doubled the length of the mouse-centric CUBIC method to 20 days to improve clearing in rats (Woo et al., 2018); Branch and colleagues successfully cleared entire rat brains by splitting samples into two hemispheres before clearing, together with increasing the timing of stages of the mouse iDISCO protocol (Branch et al., 2019); and a third method, FluoClearBABB, cleared the majority of tissue from an intact rat brain in 8 days while controlling the pH during dehydration, which, similar to uDISCO, preserved fluorescence, though the clearing at the center of the tissue is unclear from their manuscript (Stefaniuk et al., 2016).

Similar advances in imaging techniques allow for faster image acquisition. Typically, slices are imaged on confocal or 2-photon microscopes, which use point scanning methods that scan across and through tissues to excite and observe fluorescence. In contrast, light-sheet microscopy images planes instead of points, decreasing the time needed to go through the same volume of tissue. Like other methods key to modern developments, light-sheet microscopy also was first proposed in 1902 (Siedentopf and Zsigmondy, 1902), but recent developments in CMOS sensors that can acquire large images quickly, as well as advances in light sculpting (Keller et al., 2008), moving illumination and detection (Ahrens et al., 2013), and others [see review Hillman et al. (2019)] were key in making whole-brain imaging available to neuroscientists.

Inspired by these advances, we set out to produce two reliable, worker-friendly protocols for efficient clearing of intact rat brains, based on the uDISCO and iDISCO protocols. Coupled with light-sheet imaging, we can now image intact rat brains, including subcortical structures. We use these clear brains to form a light-sheet-based rat brain template for the rat neuroscience community.

**Whole-brain clearing of *Rattus norvegicus* brains**

**Figure 1.**
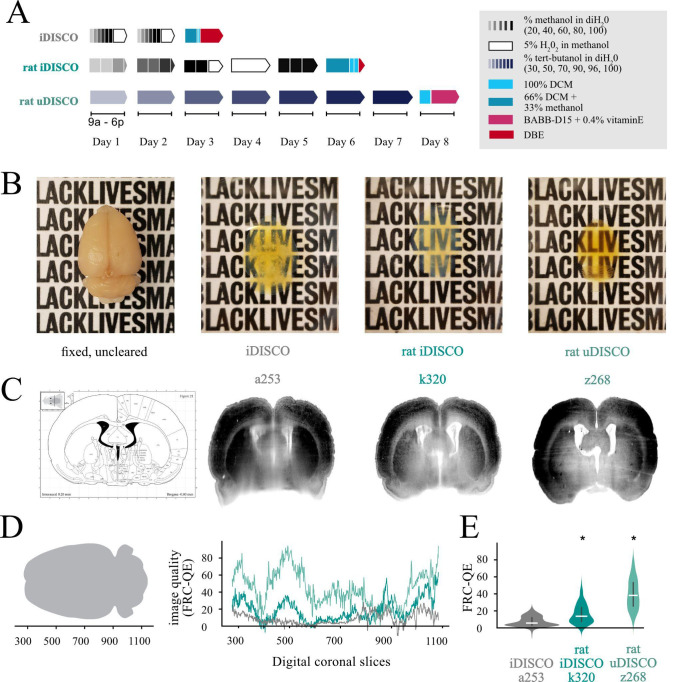
Modifications to iDISCO and uDISCO protocols allow for fully cleared rat brains. A. Schematics of iDISCO protocol and the modified rat iDISCO and uDISCO protocols (abbreviations DCM, BABB, and DBE all refer to solutions in Recipes). B. Example images of brains before clearing and after clearing with each protocol from the schematic. The uncleared brain is approximately 2.5 cm long and 1.7 cm wide. All images were taken on the same piece of paper for easy comparison. C. Computational slices through each cleared brain in B. Labels (a253, k320, z268) reflect the animal’s name. D. Drawing showing the orientation of the brain (left) and the FRC-QE scores (right) for each brain in B along the coronal axes in *slice* units. E. Quantification of the values in (D), with a Bonferroni-corrected *t*-test. Horizontal white bars are median values, and the violin limits are the 10% (bottom) and 90% (top) of the data. Asterisks indicate significance p < 0.05 compared with iDISCO a253.

We first set out to improve existing clearing techniques to work consistently on whole adult *Rattus norvegicus* brains of any size or sex. Inspired by many, we primarily focused on the DISCO techniques and the timing of stages, as this was the most effective in our preliminary explorations. We successfully made a worker-friendly protocol (Figure 1A, maximum 8 h days, compared with 10–16 h days in other protocols) that also resulted in consistently clear brains, visible in the field-standard qualitative evaluations (Figure 1B–1C) and a newer quantitative evaluation method, Fourier ring correlation–based quality estimation (FRC-QE) (Figure 1E) (Preusser et al., 2021). When applied appropriately, FRC-QE allows for comparable measurements across experiments and provides quantitative data in addition to the field-standard qualitative data.

**Princeton RAtlas**

**Figure 2.**
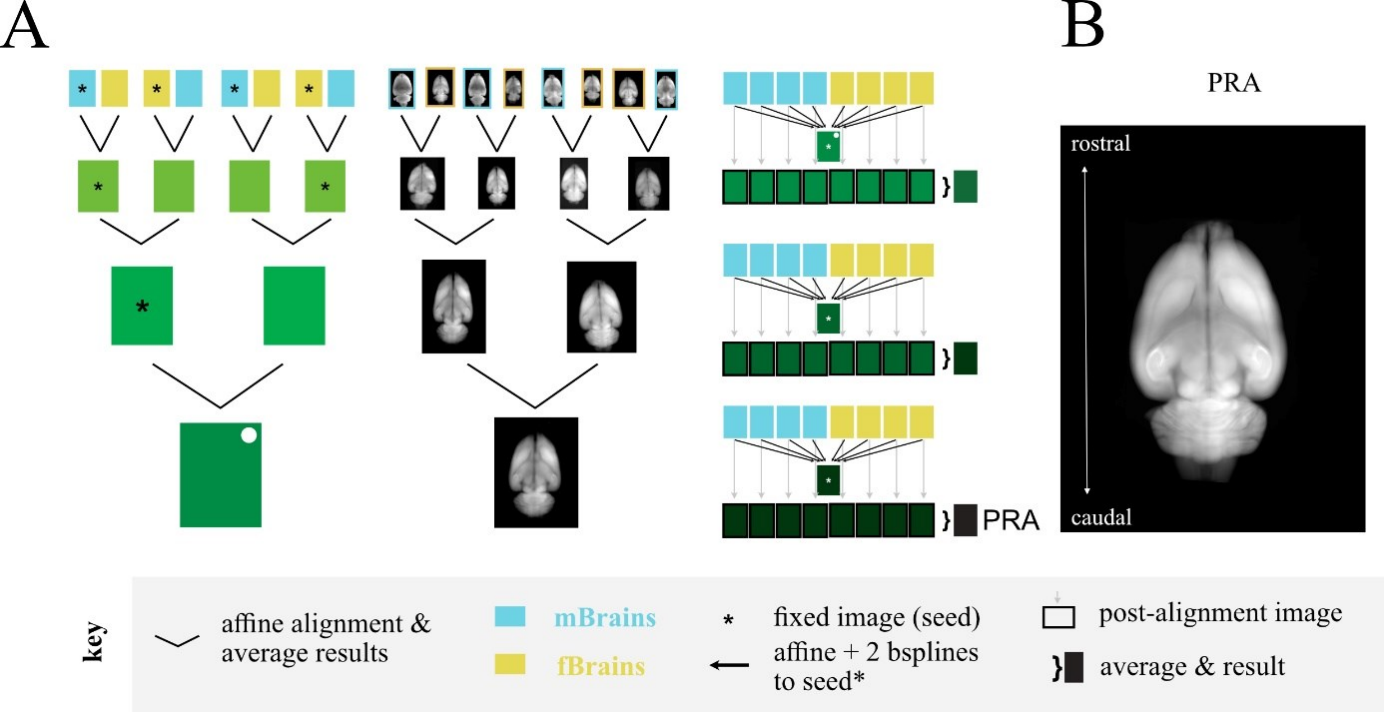
Creation of the Princeton RAtlas (PRA). A. Schematics showing the paired alignments to create the seed brain (left, middle) and the multilevel refinement resulting in the PRA (right). B. Summed axial projection of the PRA. mBrains and fBrains are male and female brains respectively, defined by external genitalia.

Once we could reliably obtain fully cleared brains, we set out to make a common coordinate framework for rats using whole-brain, light-sheet imaging. We imaged 18 brains and chose the best eight for an averaged template brain. We chose these eight based on a manual inspection of quality and based on external genitalia: we chose four male and four female brains without obvious damage or imaging aberrations, like streaking. The four female brains were all of sufficient quality that we did not need to procure additional brains, and we chose four of the male brains to match our number of females. We used an iterated, paired averaging scheme to create the seed brain (Figure 2A) and then aligned each brain to this seed, averaged the result to create the new seed, and repeated this process two additional times. The final averaged result of all brains aligned to this final seed brain became the Princeton RAtlas (PRA, Figure 2B). This coordinate space is agnostic to the sex of the animal. We also provide mPRA, a common coordinate space using only animals with visible penis and testes, and fPRA, a common coordinate space using only animals without visible penis or testes.

## Materials and reagents

**Biological materials**

1. Long-Evans rats [Hilltop Lab Animals Inc., Long Evans Hla(LE)CVF, Scottdale, PA] of any sex. Animals used in this study were 3–12 months and fed ad libitum from 290 to 597 g, though older and larger animals have since been successfully cleared but are not included in this focused manuscript.

**Reagents**

1. Ketamine (MedVet International, catalog number: RXKETAMINE, CAS: 6740-88-1)
2. Xylazine (MedVet International, catalog number: RXXYLAZINE, CAS: 7361-61-7)
3. Isoflurane (shopmedvet.com, catalog number: RXISO-100)
4. Phosphate buffered saline (PBS) (Thermo Fisher Scientific, catalog number: 14190136)
5. Heparin (Patterson Veterinary, catalog number: 07-805-8262, CAS 9005-49-6)
6. 4% paraformaldehyde in PBS (Fisher Scientific, catalog number: AAJ19943K2)
7. Methanol (Carolina Biological Supply, catalog number: 874195, CAS: 67-56-1)
8. 30% Hydrogen peroxide (Sigma-Aldrich, catalog number: H1009, CAS: 7722-84-1)
9. Dichloromethane (DCM) (Millipore-Sigma, catalog number: 270997-2L, CAS: 75-09-2)
10. Benzyl ether (DBE) (Sigma-Aldrich, catalog number: 108014-1KG, CAS: 103-50-4)
11. Vitamin E (A17039 DL-alpha-Tocopherol, 97%+) (Alfa Aesar, catalog number: 10191-41-0, CAS: 10191-41-0)
12. Benzyl alcohol (Sigma-Aldrich, catalog number: W213705, CAS: 100-51-6)
13. Benzyl benzoate (Sigma-Aldrich, catalog number: B9550, CAS: 120-51-4)
14. Diphenyl ether (DPE 99%) (A15791, 101-84-8, Alfa Aesar, Lancashire, UK, CAS: 101-84-8)
15. Tert-butanol (Sigma-Aldrich, catalog number: 471712, CAS: 75-65-0)
16. (Optional) Sodium azide (Sigma-Aldrich, catalog number: S2002, CAS: 26628-22-8)

**Solutions**

1. Methanol/diH_2_O (see Recipes)
2. 70% ethanol (see Recipes)
3. Peroxide and methanol solution (see Recipes)
4. Dichloromethane (DCM) 66%/33% Methanol (see Recipes)
5. Tert-butanol in diH_2_O (see Recipes)
6. Benzyl alcohol:benzyl benzoate (BABB) (see Recipes)
7. BABB-D15 (see Recipes)
8. 1× PBS (see Recipes)

**Recipes**

Recipes can be scaled up to clear multiple brains in parallel. The volumes below are for a single brain.

1. **Methanol/diH_2_O series**
  Make these fresh the day of or the day before, and allow them to reach room temperature, which can take 1.5 h.
  **Table:** 

Reagent	Final concentration	Volume
Methanol	20%, 40%, 60%, 80%	4 mL, 8 mL, 24 mL, 16 mL
diH_2_O	n/a	16 mL, 12 mL, 8 mL, 4 mL
Total	n/a	20 mL
2. **70% ethanol (higher concentrations are ok, used to wipe down and remove DBE)**
  This can be made ahead and stored for up to a year if in a sealed container.
  **Table:** 

Reagent	Final concentration	Volume
Ethanol (absolute)	70%	700 mL
H_2_O	n/a	300 mL
Total	n/a	1,000 mL
3. **Peroxide and methanol solution**
  This can be made ahead and stored for up to a year if in a sealed container.
  **Table:** 

Reagent	Final concentration	Volume
30% Hydrogen Peroxide (H_2_O_2_)	5%	4 mL
Methanol	80%	20 mL
Total	n/a	24 mL
4. **66% Dichloromethane (DCM) and 33% methanol solution**
  **CAUTION:** Use double or chemical nitrile gloves, work under hood or snorkel, expel slowly. This should be made within 24 h and not stored long term because of its volatility.
  **Table:** 

Reagent	Final concentration	Volume
Dichloromethane	66%	16 mL
Methanol	33%	8 mL
Total	n/a	24 mL
5. **Tert-butanol solutions**
  Make within 24 h and ideally 1.5 h ahead of use. Allow to reach 37 °C, which can take 1.5 h. In some labs, room temperature is too cold for tert-butanol, and it can solidify at higher concentrations. If so, bring up to 37 °C to make dilutions and for use either in an incubator or water bath. If your lab is too cold for liquid tert-butanol, also bring the brains up to 37 °C as described in the procedure.
  **Table:** 

Reagent	Final concentration	Volume
Tert-butanol	30%, 50%, 70%, 80%, 90%, 96%	7.2, 12, 16.8, 19.2, 21.6, 23.04 mL
diH_2_O	n/a	16.8, 12, 7.2, 4.8, 2.4, 0.96 mL
Total	n/a	24 mL
6. **BABB (Benzyl alcohol benzyl benzoate)**
  This can be made ahead and stored for up to a year if in a sealed container. Other groups have successfully replaced BABB with ethyl cinnamate (Henning, 2019), which is less toxic.
  **Table:** 

Reagent	Final concentration	Volume
Benzyl alcohol	33.33%	6 mL
Benzyl benzoate	66.66%	12 mL
Total	100%	18 mL
7. **BABB-D15**
  This can be made ahead and stored for up to a year if in a sealed container.
  **Table:** 

Reagent	Final concentration	Volume
BABB (see above)	93.38%	15 mL
Diphenyl ether (DPE)	6.22%	1 mL
Vitamin E	0.4%	0.064 mL
Total	n/a	16.064 mL
8. **1× PBS**
  This can be made ahead and stored for up to a year if in a sealed container. Recommend adding 0.1% sodium azide and/or storing at 4 °C if possible, but not required.
  **Table:** 

Reagent	Final concentration	Volume
10× Phosphate Buffered Saline	10%	50 mL
diH_2_O	90%	450 mL
Total		500 mL

**Laboratory supplies**

1. Rongeurs (Fine Science Tools, catalog number: 16022-15)
2. 20 mL scintillation vials (Millipore Sigma, catalog number: Z190535)
3. 3D-printed holder (we used a Form3 SLA 3D-printer and Black Resin from Formlabs, catalog number: RS-F2-GPBK-04) https://github.com/emilyjanedennis/PRA/blob/main/rat_brainholder.STL
4. Objective MI Plan 1.1×, NA 0.1 (Miltenyi Biotec, catalog number: 150-000-493)
5. Filter (Semrock, catalog number: FF01-525/39-25)
6. 5 mL Eppendorf tubes (Genesee Scientific, catalog number: 86-899SC)
7. Super glue. We used Loctite Super Glue (Loctite, catalog number: 212111) but any similar product should be acceptable.

## Equipment

1. Rocker (Genesee Scientific, model: 27-529)
2. Ultramicroscope II (LaVision Biotec. Bielefeld, Germany)
3. LifeCanvas SmartSPIM (LifeCanvas SmartSPIM, Cambridge, MA)
4. Fume hood/snorkel
5. Perfusion equipment. This should not be sensitive to brand or type. If you see a lot of capillaries in your cleared brains, this usually means that the perfusion is incomplete. We used MasterFlex L/S (NA5183975, VWR, Radnor, PA) and tubing size 16 (3.10 mm ID). Please also see Gage et al. (2012)
6. Access to a workstation computer and/or a computational server or cluster. There are no specific requirements, but the more powerful the computer, the faster each step will take. We recommend at least an Intel i7 Processor and 64 GB of available RAM or equivalent. More RAM, processing cores, and faster processor speeds will produce noticeable improvements
7. Waxholm Space Atlas (WHS) https://www.nitrc.org/projects/whs-sd-atlas from Papp et al. (2014)

## Software and datasets

1. ImSpector Microscope controller software Version 5.1.347 (free)
2. Abberior Instruments Development Team, Imspector Image Acquisition & Analysis Software v16.3, http://www.imspector.de
3. Elastix 4.8 or 5.0 (free)
4. Custom software (free) http://www.github.com/emilyjanedennis/PRA Scripts are written in Python (3.0+). The server-side implementation is also written in bash (2.0+)

## Procedure

- **Tissue preparation**
  There is nothing unique about this section compared to methods common in most rodent neuroscience laboratories. Please see Gage et al. (2012) for a detailed protocol with videos. There is no particular peristaltic pump required; modify the general recommendations below based on the manufacturer’s instructions for your pump.
  1. Prepare animal and environment
    - Weigh the animal.
    - Prepare 1× PBS for flushing the system, fixative solution, as well as an anesthetic cocktail based on your animal use protocol. We used ketamine (80 mg/kg) and xylazine (10 mg/kg).
    - Flush your peristaltic pump with PBS. We use PBS. **CAUTION:** Ensure no bubbles are visible in any of the tubing. If you see any bubbles, continue to flush until all bubbles are gone. Stop the flow of PBS and ensure that you can see a protruding meniscus is at the end of the tube before transferring the intake tube to the fixative solution to avoid a bubble.
  2. Perfuse animal (at room temperature)
    - Anesthetize the animal based on your animal care requirements. We used 3% isoflurane in oxygen. Once the animal is asleep, administer the anesthetic cocktail. Wait and ensure the animal is in a deep, surgical plane of anesthesia following your animal care requirements.
    - Make an incision under the ribcage across the animal’s abdomen. Separate the liver and diaphragm. Cut the diaphragm. Then, cut the ribs on either side. Be careful not to harm the heart or lungs; use a hemostat to hold the ribcage and slowly pull up and away from the body towards the head to expose the heart and lungs.
    - Insert a 15 gauge needle into the posterior left ventricle. You may snip the area with scissors if the needle does not easily puncture the ventricle. You may clamp the needle in place, either over the aorta or at the incision site.
    - Use scissors to snip the animal’s right atrium only.
    - Begin perfusing PBS through the animal. Adjust the needle if needed [see Gage et al. (2012) and troubleshooting tip 1 for details]. Run until the liver loses its color and the fluid exiting the right ventricle is clear; this should be approximately 200 mL.
    - Switch the perfusion solution from PBS to fixative. The animal may twitch; this is expected. After 200 mL of fixation fluid, the animal should feel stiff all over. You can make your own fixative solution, but we purchased 4% paraformaldehyde in PBS.
  3. Extract brain
    - Remove the head with scissors or a guillotine.
    - Use scissors to trim muscles and to expose the skull by making an incision from the neck to the face.
    - Carefully remove the skull with rongeurs. If the skull chips into pieces, the edges can damage your sample. Take care to avoid this.
    - Use a spatula, a surgical blade, or small scissors to sever the nerves around the edges of the brain until it comes free.
    - Place brain in fixative solution in a 15–20 mL Eppendorf or scintillation vial overnight (10–24 h). Use vials made of either glass or polypropylene.
  4. Wash brain
    - Wash brain with 1× PBS three times for 30 min each, rocking or shaking at room temperature.
    - **OPTIONAL:** Samples can be stored in PBS for 3 days or less at 4 °C.
- **Tissue processing (uDISCO, Figure 1A)**
  You should follow *either* A (uDISCO) or B (iDISCO, below) but not both. To transfer the brains to a new solution, you may reuse the same vial throughout unless otherwise noted. To do this, we recommend decanting the current solution without decanting the brain, then filling the vial with the next solution until the brain is floating and completely covered. We used glass scintillation vials, but polypropylene is also acceptable.
  1. Dehydration
    - Mix tert-butanol solutions to allow them to reach room temperature (~1.5 h).
    - 5 min rocking at room temperature in 15–20 mL Eppendorf tubes or scintillation vials in 0.1 M PBS. Use vials made of either glass or polypropylene.
    - Transfer brain to 30% tert-butanol, rocking at 37 °C overnight (10–24 h).
    - Transfer brain to 50% tert-butanol, rocking at 37 °C overnight (10–24 h).
    - Transfer brain to 70% tert-butanol, rocking at 37 °C overnight (10–24 h).
    - Transfer brain to 80% tert-butanol, rocking at 37 °C overnight (10–24 h).
    - Transfer brain to 90% tert-butanol, rocking at 37 °C overnight (10–24 h).
    - Transfer brain to 96% tert-butanol, rocking at 37 °C overnight (10–24 h).
    - Transfer brain to 100% tert-butanol, rocking at 37 °C overnight (10–24 h).
  2. Clearing
    - Transfer brain to 100% DCM, rocking at room temperature for 2.5–3 h. **CAUTION:** Wear double nitrile gloves and work under a ventilation hood or snorkel.
    - Carefully wipe with 70% (or higher) ethanol in water to remove any solution on the exterior of the vial or tube.
    - Transfer brain to a fresh final vial with BABB-D15 for three or more hours. Inverting (not rocking) several times (ideally every 30 min but can be flexible between 15–45 min).
    - Carefully wipe with 70% (or higher) ethanol in water to remove any solution on the exterior of the vial or tube.
    - Cleared brains can be stored indefinitely at room temperature or 4 °C. We recommend 4 °C for long-term storage.
- **Tissue processing (iDISCO, Figure 1A)**
  You should follow either A (uDISCO, above) or B (iDISCO) but not both. To transfer the brains to a new solution, you may reuse the same vial throughout unless otherwise noted. To do this, we recommend decanting the current solution without decanting the brain, then filling the vial with the next solution until the brain is floating and completely covered. We used glass scintillation vials, but polypropylene is also acceptable.
  1. Dehydration
    - Mix fresh methanol solutions to allow them to reach room temperature (~1.5 h).
    - Transfer brain to 20% methanol in diH_2_O rocking at room temperature for 3 h.
    - Transfer brain to fresh 20% methanol in diH_2_O rocking at room temperature for 3 h.
    - Transfer brain to 40% methanol in diH_2_O rocking at room temperature for 2 h.
    - Transfer brain to 40% methanol in diH_2_O rocking at room temperature overnight.
    - Transfer brain to 60% methanol in diH_2_O rocking at room temperature for 3 h.
    - Transfer brain to 60% methanol in diH_2_O rocking at room temperature for 3 h.
    - Transfer brain to 80% methanol in diH_2_O rocking at room temperature for 2 h.
    - Transfer brain to 80% methanol in diH_2_O rocking at room temperature overnight.
    - Transfer brain to 100% methanol rocking at room temperature for 3 h.
    - Transfer brain to fresh 100% methanol rocking at room temperature for 3 h. Can be stored for months in 100% methanol at 4 °C in the dark without rocking.
  2. Bleaching
    - 48–72 h in peroxide and methanol solution (5% H_2_O_2_ in methanol) at room temperature with rocking.
    - Wash brain three times in 100% methanol rocking at room temperature for 2 h each wash. Can be stored for months in 100% methanol at 4 °C in the dark without rocking.
  3. Clearing
    - Transfer brain to 66% DCM/33% methanol rocking at room temperature for 6 h (or up to 8 h). **CAUTION:** Wear double nitrile gloves and work under a ventilation hood or snorkel when working with DCM. Wipe outside of tube or vial with 70% or greater ethanol.
    - Transfer brain to 100% DCM rocking at room temperature for 1 h and wipe outside of tube or vial with 70% or greater ethanol.
    - Transfer brain to fresh 100% DCM for 1 h.
    - Transfer brain to fresh 100% DBE at room temperature without rocking for 3–24 h. When possible, invert sample several times.
    - Transfer brain to a fresh tube or vial of 100% DBE at room temperature and wipe outside of tube or vial with 70% or greater ethanol.
    - Samples can be stored indefinitely in DBE at room temperature or 4 °C without rocking. We recommend 4 °C in the dark for long-term storage and ensuring minimal air in the vial.
- **Imaging the sample: Imaging details will depend on your microscope and experimental goals**
  Examples could include using higher magnification (at least 4×) for counting labeled cell centers, or lower (1.1×) for general imaging of gross anatomy to, for example, make a common coordinate system or identify a probe’s location. You will likely need to use *tiles* to cover the entire brain. We recommend at least 20% overlap for all tiles to assist the fusion and alignment process later. Also see Pisano et al. (2022).
  1. Set up the microscope with appropriate filters. For autofluorescence, shorter wavelengths provide more information. We recommend 488 nm excitation, 525 nm emission (or lower wavelengths). Ensure the light-sheets are aligned according to the manufacturer’s specifications. This process will be slightly different for every microscope, so please check your manual before proceeding.
  2. Don gloves and ensure you are not using any plastic items unless you are sure they are compatible with the chemicals in use.
  3. Add the refractive index matched solution (BABB-D15 for uDISCO or DBE for iDISCO) to the microscope chamber according to the manufacturer’s instructions. Take care, as some microscopes will not be compatible and either solution could melt some plastics.
  4. Clean the brain holder (custom 3D-printed or manufacturer-provided) with ethanol and let dry. Place a cleared brain onto a paper towel, then use the smallest possible amount of super glue to affix the brain to the holder. We typically super glue either the brainstem or olfactory bulbs, depending on project needs (Figure S2).
  5. Insert the brain into the solution in the microscope.
  6. Image the brain: turn on lasers, camera, stage, and software. We recommend using 20% overlap or greater for tiles. We also recommend imaging with the smallest axis parallel to the detection axis (the objective) and perpendicular to the light-sheets (such that the left light-sheet enters the left side of the brain, the right light-sheet enters the right side of the brain, and the objective images the *z-axis* from dorsal to ventral).
  7. After acquisition, remove the brain, and replace it back in DBE or BABB-D15. Power off all items, clean thoroughly.
  8. Some imaging platforms will automatically blend the light-sheets or stitch the tiles to create single z planes. Often, this is sufficient, but acquiring raw data before blending and stitching is recommended in case these steps are suboptimal and need to be repeated.
- **Create a 3D volume: there are many tools available for these steps**
  We provide free code to handle images from either LaVision Ultrascope II or SmartSPIM microscopes. However, the code can be altered to allow for alternative inputs.
  1. On a cluster, run either:
    - spim_downsize_n_register.sh providing the required inputs: the path to the main image folder, the registration channel subfolder name, and the cell channel subfolder name. If you only have one channel imaged, just repeat the registration channel folder name.
    - sub_registration.sh
- **Align to the Princeton RAtlas**
  This section assumes you have already installed all the necessary software. Detailed instructions are available in the GitHub repository README.

## Data analysis

**Figure 3.**
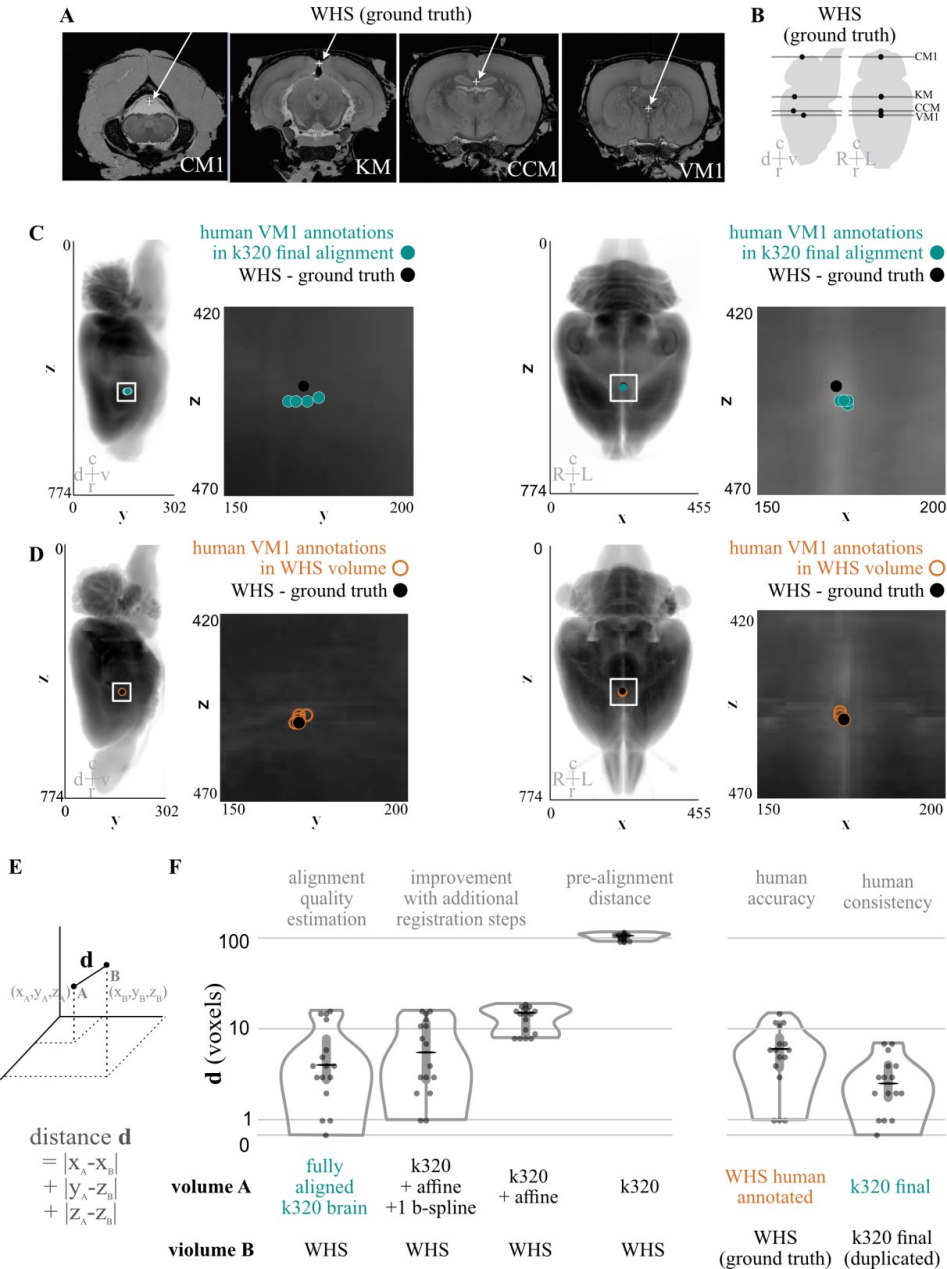
Quantification of computational alignment efficacy compared with humans. A. Slices from the Waxholm Space Atlas of the Adult Rat Brain demonstrating the location (white arrow, +) of identifiable points of interest. Abbreviations: d/v dorsal/ventral, c/r caudal/rostral, L/R left/right. B. Drawing of the location of each slice from A shown in sagittal and axial spaces. C. Sagittal (left) and axial (right) examples of four human annotators’ points for location VM1 (ventricle middle 1) in a single brain compared with imported and aligned ground truth data from the Waxholm atlas. D. Sagittal (left) and axial (right) examples of four human annotators’ points for location VM1 made in Waxholm atlas space, compared with the ground truth data from the atlas. E. Simplified diagram demonstrating how to calculate the distance metric used in F. F. Quantification of distances between user-annotated points in distinct stages of alignment and the imported annotations (WHS). G. Quantification of distances demonstrating the accuracy (left) and precision (right) of human annotators for all four annotated points.

We quantified the opacity of our cleared tissues in two ways: the field-standard qualitative assessment of reading text through a cleared brain (Figure 1B), and quantitatively, using FRC-QE (Figure 1D–1E) (Preusser et al., 2021). Next, inspired by the Scalable Brain Atlas approach (Bezgin et al., 2009; Sergejeva et al., 2015), we quantified the process of importing annotations into the Princeton RAtlas (PRA) space by asking four experienced humans to annotate points in anonymized volumes (Figure 3). These volumes included a cleared brain (k320) pre-alignment, after affine alignment, after affine and 1 b-spline alignment, and after affine and 3 b-spline alignment (final alignment) to the atlas. Details on these alignment types can be found in Figure S1. We also included a volume containing the Waxholm Space Atlas (WHS) to measure the accuracy of our human annotators and, unbeknownst to them, a second copy of the fully aligned k320 brain to quantify their precisions.

## Validation of protocol

In addition to the eight animals used to create the PRA, we discarded two brains due to damage or streaking in the imaging, and six additional successfully cleared brains were used for supplementary figures and further internal validation on various sizes of brains.

## General notes and troubleshooting

1. Waste disposal. Please refer to your local requirements and consult with your Environmental Health and Safety or appropriate agency. In New Jersey, where these experiments were conducted, all reagents except PBS and water are considered hazardous waste and should be handled with care. Aside from PBS and water, none of these reagents should be discarded in a standard sink.
2. Perfusion: The quality of the perfusion can strongly impact the clearing quality. For example, if you see lots of autofluorescence that look like capillaries in your final cleared brains when imaging, this is often a sign of a bad perfusion. See Gage et al. (2012) for details on how to properly perfuse, and also the specific notes below for common problems.
  - If the heart stops before needle insertion, clots will begin to form. Practice until you can quickly dissect and insert the needle while the heart is still bleeding.
  - During perfusion, if you see bloody discharge from the nose or mouth of the animal, you have likely inserted the needle incorrectly. Check that the needle is in the left atrium but do not insert too deep as you can puncture the internal wall between the atria.
  - During perfusion, if anything is punctured except the left ventricle, attempt to clamp the wound.
3. Installing Elastix 5.0 is often the hardest step of the analysis.
  - On Windows, follow the instructions on “read the docs” https://simpleelastix.readthedocs.io/GettingStarted.html#compiling-on-windows starting with step 3, the command line, and skip the IDE in steps 4 and 5. If the Python wrapping step fails, see this known issue https://github.com/SuperElastix/SimpleElastix/issues/243.
  - On Linux or MacOS: follow the instructions in the Elastix 5.0 manual under “the easy way” and not the “super easy way.” Ensure you have ITK 5.0.

## Supplementary information

The following supporting information can be downloaded here:

1. Figure S1. Demonstration of the selection process for parameter files
2. Figure S2. Example diagram of glued brains
